# Identification of novel QTLs and development of KASP markers for sugar content in fresh and dried peanut (*Arachis hypogaea* L.)

**DOI:** 10.3389/fpls.2025.1745366

**Published:** 2026-01-22

**Authors:** Chi Zhang, Mingshuo Liu, Shunli Cui, Miao Chen, Hongtao Deng, Lifeng Liu, Yingru Liu, Xiukun Li, Xiaoshu Chen, Mingyu Hou

**Affiliations:** 1 State Key Laboratory of North China Crop Improvement and Regulation, Key Laboratory for Crop Germplasm Resources of Hebei, Hebei Agricultural University, Baoding, China; 2 North China Key Laboratory for Crop Germplasm Resources of Education Ministry, College of Agronomy, Hebei Agricultural University, Baoding, China; 3 Peanut Institute, Jilin Academy of Agricultural Sciences (Northeast Agricultural Research Center of China), Changchun, China

**Keywords:** drying maturation seed, fresh seed, KASP, peanut, QTL, sugar content

## Abstract

The total sugar content (TSC), soluble sugar content (SSC), and sucrose content (SC) are key determinants of taste and flavor of fresh and dried peanut kernels, and are important quality indicators in peanut breeding. However, the quantitative trait locus (QTLs) regulating peanut sugar content, especially in fresh seeds, remain poorly understood. In this study, TSC, SSC, and SC were measured in dried mature seeds (DMS) across four environments (three-year data from Baoding and Fuxin) and in fresh seeds (FS) across two environments (Baoding and Fuxin), and QTL mapping was performed in a recombinant inbred line (RIL) population (‘Silihong’×’Jinonghei3’). TSC, SSC, and SC were all lower in FS compared to DMS, indicating that the sugar content increased during the drying and maturation process. Two major co-localized QTLs, *qA06* (physical location 115.08-115.73 Mb) and *qB06* (physical location 147.74-148.46 Mb), were identified in multiple environments. *qA06* was associated with TSC, SSC, and SC in DMS, and SSC in FS, spanning a 0.65 Mb physical interval. *qB06*, spanning a 0.72Mb physical interval, was associated with TSC, SSC, SC in DMS, and TSC, SSC in FS. *qB06* represents a newly identified QTL in this study; within 56 candidate genes and 319 SNPs were screened. Among them, the genes *arahy.3URM83* and *arahy.41Y8R9*, and *arahy.P7PTW7* were closely related to sugar synthesis. Transcriptome analysis during the drying and maturation stages revealed that *arahy.3URM83* and *arahy.41Y8R9* were strongly associated with sugar content. The QTL regions identified in this study not only elucidate the genetic regulatory mechanism of peanut sugar content under different drying conditions but also enable the development of KASP markers, offering valuable resources for peanut quality improvement and targeted breeding programs.

## 1 Introduction

The total global peanut production exceeds 40 million tons, with 55% used for oil extraction and 35% - 45% used for various forms of consumption and food processing. More than half of the peanuts used for consumption are roasted, and this proportion continues to increase each year (Wang et al., 2025). The taste of fresh and roasted peanuts is primarily determined by sugars and volatile organic compounds. In addition, the sugar content of peanuts is closely related to their stability during storage. An optimal sugar content helps maintain the vitality of peanut seeds and reduces mold risk associated with abnormal sugar metabolism (Wang et al., 2018). Therefore, understanding the genetic and physiological mechanisms underlying sugar accumulation in peanuts is of great significance for specialized peanut storage and production.

Fresh peanuts refer to peanut seeds that are harvested and used directly for cooking without drying. Significant differences exist between fresh peanuts and dried peanuts in both sugar composition and concentration. Fresh peanut seeds have a high moisture content, and sugars comprise predominantly monosaccharides such as glucose and fructose, as well as oligosaccharides such as sucrose. After drying, the physiological metabolism of peanut seeds slows down due to water loss, and the consumption of sugars by respiration decreases. In addition, physical and chemical processes such as the Maillard reaction may occur during the drying process, affecting sugar transformation and concentration. Compared with fresh seeds, the sugar content and polysaccharide proportion of dried peanuts are significantly increased, resulting in a sweeter taste (Pattee et al., 2000). There are genetic differences underlying variation in sugar content between fresh and dried peanut kernels (Deng et al., 2025), suggesting distinct regulatory mechanisms. Therefore, it is necessary to conduct in-depth research in order to generate and cultivate specialized peanut varieties and guide peanut production.

Peanut sugar content is primarily governed by genetic factors but is also significantly influenced by the environment. Adequate and suitable light can promote the efficient synthesis of photosynthetic products in peanut leaves and their transport to seeds, thereby increasing sugar content (Wu et al., 2025). In terms of temperature, during the swelling period of peanut pods, significant diurnal temperature variations promote the accumulation and conversion of sugars. Soil fertility is equally important. A soil rich in nitrogen, phosphorus, potassium, and other nutrients in a balanced proportion can provide sufficient nutrients for peanut growth, can facilitate efficient sugar biosynthesis pathways, thereby enhancing peanut sugar content (Zhou et al., 2007). Comprehensive evaluation of sugar content in genetically diverse peanut populations under varying light regimes can enable a more integrated understanding of the genetic and environmental factors regulating sugar metabolism and accumulation, and provide a theoretical basis for the production of high (low) sugar peanuts.

Sugar content in peanuts is a complex quantitative trait, controlled by multiple genes. QTL mapping is a valuable approach for understanding the genetic regulation mechanism of plant sugar content. Park et al. (2013) constructed a recombinant inbred line population and successfully identified multiple QTL loci related to sugar content, such as sucrose and glucose, in corn grains using high-density genetic mapping. Li et al. (2025) combined analyses of parental and natural populations to identify multiple QTLs related to ear sugar content in maize. Recently, the study of sucrose content regulation in peanut kernel has attracted the attention of many researchers. Current studies predominantly concentrate on the genetic mechanisms underlying sucrose accumulation in dried peanuts, with several QTLs for sucrose content in dried peanuts having been identified (Li et al., 2023; Guo J. et al., 2023; Guo J. B, et al., 2023; Wang et al., 2024, 2024). However, QTLs for TSC and SSC in dried peanuts, and sugar content in fresh peanuts remain largely uncharacterized. A systematic investigation of the sugar content and type in fresh and dried peanuts across different environments is essential for elucidating the roles of QTLs, the metabolic processes involved, and the interactions between genes and the environment.

In order to gain a deeper understanding of the genetic regulation mechanism of peanut sugar content, this study employed a RIL population exhibiting a normal distribution of sugar content-related traits, such as total sugar, soluble sugar, and sucrose. Through high-throughput sequencing and phenotypic assessment across different growth environments and developmental stages (fresh and dried seeds), QTL mapping was performed to identify loci associated with sugar accumulation. In order to clarify the key genes and genetic effects that control sugar content, expression analysis and KASP marker development were conducted on candidate genes to systematically carry out genetic analysis of peanut sugar content. These integrative analyses enhance our understanding of the genetic regulatory network of peanut sugar content, but also provide important theoretical basis and technical support for peanut quality breeding, supporting the sustainable development of the peanut industry.

## 2 Materials and methods

### 2.1 Plant materials

The RIL population, comprising 248 individual lines derived from Silihong (A. *hypogaea* var. *fastigiate*) ×Jinonghei3 (A. *hypogaea* var. *hypogaea*), was used for QTL mapping. The population was developed at the experimental station of Hebei Agriculture University, Baoding, China. The RIL population and its parental lines were planted in experimental fields in Baoding (38.79°N, 115.56°E), Hebei Province (planted in May and harvested in September of 2018 (E1), 2020 (E2), and 2021 (E3)) and Fuxin (N42°01′, E121°40′), Liaoning Province (planted in May and harvested in September of 2021 (E4)). The parental lines and their 248 RILs were grown as previously described (Li et al., 2024). The browning of the inner fruit shell color signals the optimal timing for peanut harvest (Liang et al., 2018). At physiological maturity, seeds (E1 and E2) from eight middle-row plants in each plot were harvested and air-dried. 50% of the seeds harvested from E3 and E4 were stored at -20°C for FS sugar content analysis, and 50% were naturally air dried for DMS sugar content analysis. Store for future use once moisture content drops below 8% (Qu et al., 2022).

### 2.2 Determination of sugar content in peanut seeds

The total sugar content was determined using the 3, 5-dinitrosalicylic acid method according to Hou et al. (2017). Soluble sugar content was determined by the anthrone colorimetric method, as improved by Chen et al. (2022). A 0.1 g tissue sample was extracted from the middle section of the peanut cotyledon and homogenized using a tissue grinder (JXFSTPRP-24 A, Shanghai Jing Xin Co., LTD.). Subsequent procedures were performed in strict accordance with the manufacturer’s instructions of the sugar content assay kit (Gris Biotechnology Co., LTD.).

### 2.3 Data analysis and heritability estimation

Statistical analyses, including analysis of variance (ANOVA), frequency distribution, and the independent sample t-test analysis, were performed using the IBM SPSS Statistics version 25 software (IBM SPSS, USA).

### 2.4 QTL analysis

A high-density genetic map previously constructed in our laboratory was used for QTL mapping (Li et al., 2024). The inclusive composite interval mapping (ICIM) method in QTL IciMapping V4.2 software was used for scanning the phenotypic value and sugar content data in every environment. To validate the authenticity of detected QTLs and obtain more information, LOD≥2.5 and 1000 permutation test (PT) were employed as threshold methods. The PT method was used for detecting additive QTL. For individual environment analyses, as well as multi-environment joint and epistatic analyses, the BIP and MET functional modules of QTL IciMapping v4.2 were applied. QTLs were named as *q* + the abbreviated trait name + linkage group number, designating one of multiple QTL in a single linkage group following the international Rules of Genetic Nomenclature (Li et al., 2024).

### 2.5 Expression of candidate genes during the drying process

Candidate gene expression profiles related to sugar content were generated from transcriptome data (Deng et al., 2025). Transcriptome sequencing were used to analyze the regulatory mechanism during the drying process at five time points in parents. The transcriptome data of Silihong deposited in the NCBI SRA database (accession: PRJNA1212227). The transcriptome data of Jinonghei3 was unpublished.

### 2.6 KASP maker development and validation

The SNPS in the QTL interval of Silihong and Jinonghei3 were resequenced to develop KASP markers and subsequently screened. The primers were designed based on the differential SNP sequences identified. The primer sequences of KASPs are presented in **Table 1**. KASP assays were performed in 10 μL reaction volumes comprising 2.0 μL of genomic DNA (25–50 ng), 5 μL of 2×AQP master mix, 0.15 μmol L-1 of each forward primer, 0.4 μmol L-1 of the common reverse primer, and double-distilled water (JasonGen Biotech Co., Ltd., Beijing, China). PCR was performed on an ABI QuantStudio6 machine (ABI Life Technologies, CA, USA) using the following PCR conditions: 95°C for 10 min; 95°C for 20 s, 61°C (−0.6°C per cycle) for 40 s for 10 cycles; 95°C for 20 s, 55°C for 40 s for 31 cycles. Fluorescence was detected, and data analysis was manually performed using the built-in ABI QuantStudio6 software. Then, the KASP markers were assessed in a peanut panel comprising 13 lines and 19 peanut varieties and landraces cultivated in China.

**Table 1:** KASP primers in the *qSB06* interval.

Gene	Location (bp)	Primer	Peanut genome DNA sequence
*arahy.B6Y69S*	147735746	F1	5’ GTTTCTTCTTCAAATTACCAAGAAGAT 3’
		F2	5’ GTTTCTTCTTCAAATTACCAAGAAGAG 3’
		R	5’ CTCATGGTGGCTATAGTGCTATACTCT 3’
	147735758	F1	5’ AATTACCAAGAAGATGAAGGTGTAAGG 3’
		F2	5’ AATTACCAAGAAGATGAAGGTGTAAGC 3’
		R	5’ TCAAATGGCTAGCTAGTAATACCATTG 3’
*arahy.3URM83*	148167024	F1	5’ TGGGGTTCTTAATTGTCAATTAGAAA 3’
		F2	5’ TGGGGTTCTTAATTGTCAATTAGAAC 3’
		R	5’ AGAAGTGGAAGTGTAACCAAACTTGG 3’
*arahy.PKWB63*	148038550	F1	5’ GAAGGTGACCAAGTTCATGCTCCTCGGCGGCGAGGTC 3’
		F2	5’ GAAGGTCGGAGTCAACGGATTCCTCGGCGGCGAGGTT 3’
		R	5’ CTTGGATCTTGCTCGGTTTCT 3’

## 3 Results

### 3.1 Analysis of sugar content in peanut kernel

**Figure 1:**
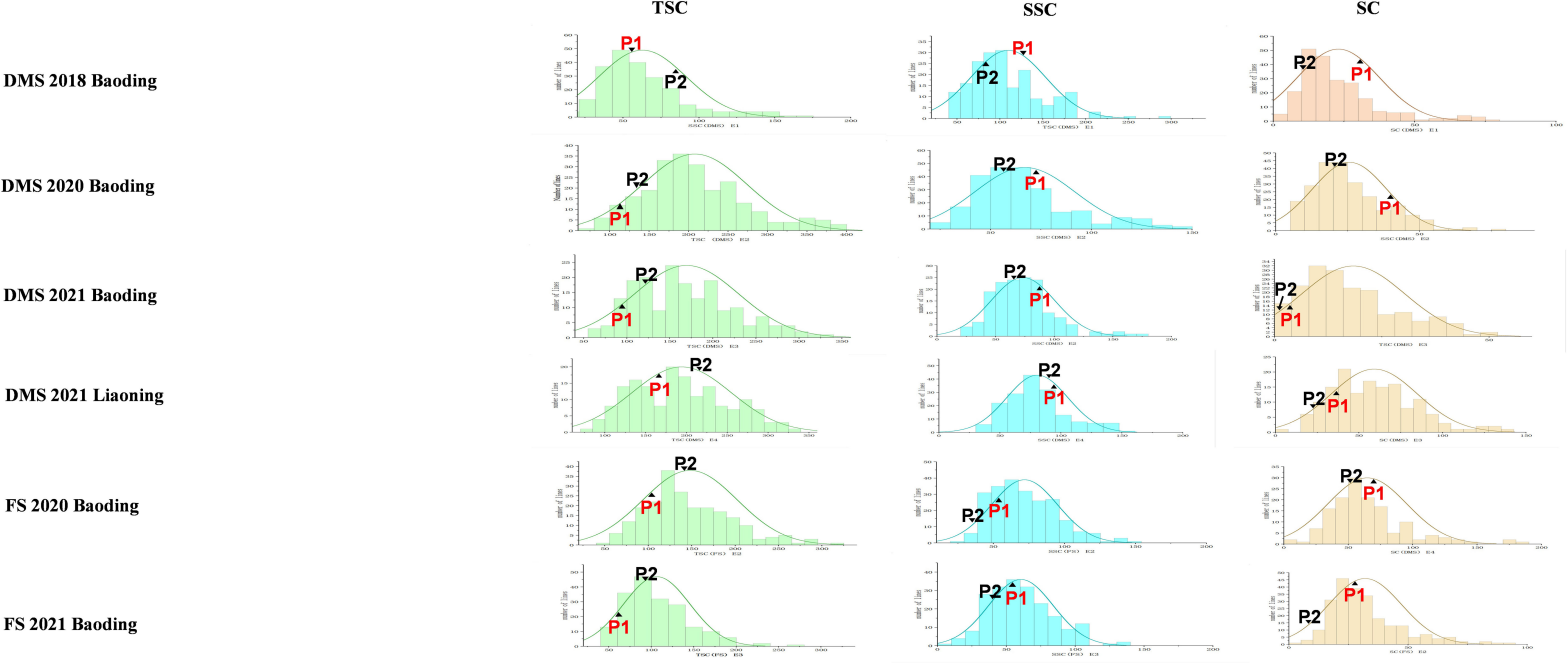
Phenotypic distribution of sugar content traits for the RIL population. The x-axis shows the range of sugar content traits, including total sugar, soluble sugar, and sucrose in six environments. The y-axis shows the number of individuals of the RIL population. P1 and P2 represent the parents ‘Silihong’ and ‘Jinonghei3’, respectively.

In both fresh seeds (FS) and dried mature seeds (DMS), the TSC of Jinonghei3 was higher than that of Silihong, and the SSC and SC contents of Silihong exceeded those of Jinonghei3. However, across conditions, TSC, SSC, and SC of lines in DMS were higher than in FS (**Figure 1**). This indicates that the drying process affects the sugar content and that there are genetic differences.

The sugar contents of FS and DMS were affected by the environment. The TSC of DMS in E1 was lower than in other environments. The coefficients of variation for SC in DMS and FS were higher than those of TSC and SSC in the corresponding environment (**Table 2**), and transgressive segregation for SC was more pronounced in the offspring population.

**Table 2:** Phenotypic variation of sugar content traits among the population in six environments.

Traits	Environment		Silihong (mg/g)	Jinonghei3 (mg/g)	Range (mg/g)	Mean (mg/g)	SD	*CV* (%)
TSC (DMS)	E1	2018BD	70.48	80.39	47.49-319.65	126.44	51.06	40.38
	E2	2020BD	147.50	205.51	64.20-397.10	207.52	65.97	31.79
	E3	2021BD	95.70	135.81	62.15-389.27	169.23	60.42	35.70
	E4	2021FX	169.64	226.16	84.74-379.55	193.74	60.78	31.37
	mean		120.83	161.96	64.64-371.39	174.23		
TSC (FS)	E3	2021BD	107.49	139.73	53.74-387.96	147.92	54.27	36.69
	E4	2021FX	85.22	121.94	41.01-294.86	109.09	43.56	39.93
	mean		96.35	130.83	47.37-341.41	128.50		
SSC (DMS)	E1	2018BD	80.73	56.16	12.92-164.00	62.69	28.72	45.81
	E2	2020BD	74.78	56.16	24.00-143.20	66.91	24.78	37.03
	E3	2021BD	37.50	36.71	22.54-172.86	73.14	27.24	37.24
	E4	2021FX	95.16	80.49	34.49-157.72	80.54	25.12	31.19
	mean		72.04	57.38	23.48-159.44	70.82		
SSC (FS)	E3	2021BD	52.19	40.00	25.54-144.42	71.75	23.23	32.38
	E4	2021FX	55.42	36.10	4.82-130.75	58.55	22.46	38.36
	mean		53.805	38.05	15.18-137.58	65.15		
SC (DMS)	E1	2018BD	28.57	12.11	2.30-76.35	22.92	14.52	63.35
	E2	2020BD	28.57	12.11	6.29-78.85	25.58	12.98	50.74
	E3	2021BD	35.57	24.09	1.71-137.68	58.98	25.81	43.76
	E4	2021FX	67.83	55.88	2.32-122.99	58.39	21.64	37.06
	mean		40.13	26.04	3.15-103.96	41.46		
SC (FS)	E3	2021BD	30.40	10.93	4.62-90.97	31.99	15.24	47.64
	E4	2021FX	5.73	4.69	1.11-62.17	20.32	12.06	59.35
	mean		18.06	7.81	2.86-76.57	26.15		

TSC, SSC, and SC were all affected by both environmental and genetic factors. Heritability estimates indicated that genetic control predominated. TSC, SSC, and SC exhibited relatively high broad-sense heritability (*h^2^*), ranging from 0.66 to 0.89 (**Table 3**). Through genetic analysis of multi-environmental populations, the loci associated with phenotypic variation could be analyzed, and then the genetic mechanism of TSC, SSC, and SC in peanut was studied.

**Table 3:** Analysis of the broad-sense of heritability of sugar content in peanut.

Trait	Source	DF	SS	MS	F-value	P-value	*h*^2^ (%)
TSC (DMS)	Environment	3	309710.258	103236.753	40.621	<0.001	80
	Genotype	245	3395499.106	13859.180	5.453	<0.001	
	Environment×Genotype	409	3834430.892	9375.137	3.689	<0.001	
	Error	1660	4218777.660	2541.432			
TSC (FS)	Environment	1	115533.347	115533.347	82.883	<0.001	85
	Genotype	243	780375.845	3211.423	2.304	<0.001	
	Error	172	239757.388	1393.938			
SSC (DMS)	Environment	3	122615.494	40871.831	132.937	<0.001	83
	Genotype	245	723648.409	2953.667	9.607	<0.001	
	Environment×Genotype	586	1304829.923	2226.672	7.242	<0.001	
	Error	1323	14586.458	307.453			
SSC (FS)	Environment	1	13308.506	13308.506	26.337	<0.001	82
	Genotype	240	138585.146	577.438	1.143	0.168	
	Error	191	96514.343	505.311			
SC (DMS)	Environment	3	388137.34	3.000	129379.113	<0.001	89
	Genotype	243	220654.977	243.000	908.045	<0.001	
	Error	590	250344.584	590.000	424.313		
SC (FS)	Environment	1	16752.436	16752.436	95.540	<0.001	66
	Genotype	244	50514.910	207.028	1.181	0.120	
	Error	178	31211.437	175.345			

### 3.2 Analysis of QTLs associated with sugar content in peanut kernels

#### 3.2.1 QTLs associated with the sugar content of DMS

**Figure 2:**
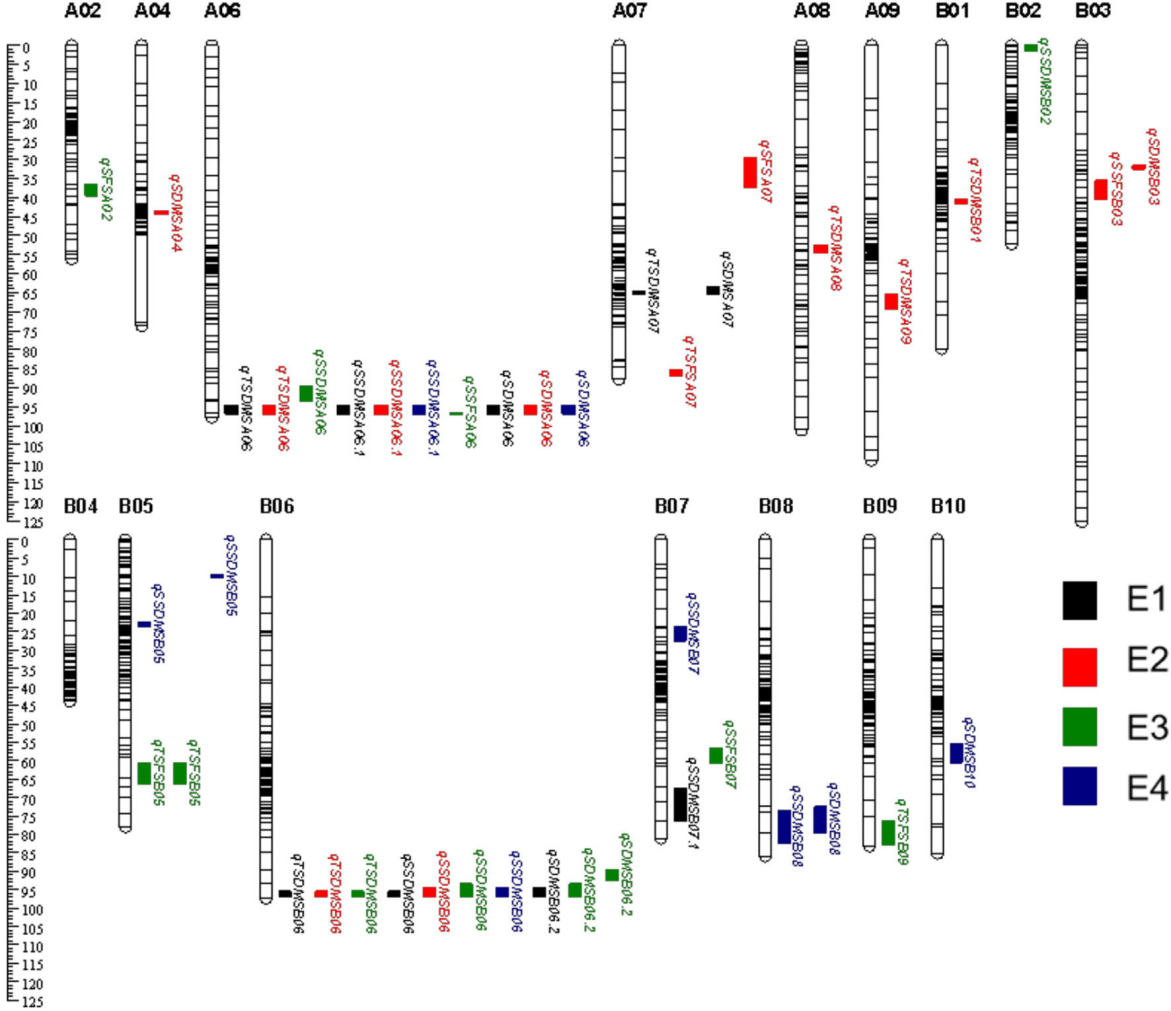
Distribution of QTL related to sugar content in four environments was identified by using composite interval mapping (CIM).

A total of 22 QTLs associated with sugar content were detected in DMS. 6 QTLs associated with TSC-DMS with PVE of 2.75%-18.03% were mapped on 6 chromosomes, namely A06, A07, A08, A09, B01, and B06 (**Figure 2**). *qTSDMSA06* and *qTSDMSB06* were stably expressed across several environments with PVE between 7.69%-18.03%. The other 4 QTLs were only detected in a single environment (**Table 4**).

**Table 4:** Identified QTLs for TSC, SSC, SC in DMS and FS of peanut^(1)^.

Traits^(2)^	QTL	Env.^(3)^	Chr.	Pos. (cM)	Maker interval	P.I. (bp)	LOD	PVE (%)	Add.
TSC(DMS)	*qTSDMSA06*	E1	A06	96	1066-1067	115,079,3255-115,729,525	13.51	17.87	24.69
		E2	A06	97	1067-1068	115,729,525-116,234,709	7.62	7.69	21.43
	*qTSDMSA07*	E1	A07	65	1179-1181	67,339,371-68,712,294	2.71	3.10	-9.73
	*qTSDMSA08*	E2	A08	53	1250-1251	33,205,452-33,827,201	3.41	3.35	-13.43
	*qTSDMSA09*	E2	A09	67	1475-1476	112153625-112825494	3.36	3.18	-13.02
	*qTSDMSB01*	E2	B01	41	1932-1938	104404515.5-107138843	2.91	2.75	-12.13
	*qTSDMSB06*	E1	B06	97	3330-3331	147741044-148463751	10.78	14.02	-20.94
		E2	B06	97	3330-3331	147741044-148463751	16.51	18.03	-31.59
		E3	B06	97	3330-3331	147741044-148463751	3.55	7.81	-17.49
TSC(FS)	*qTSFSB05*	E3	A05	63	876-877	111814557.5-112520508	2.82	6.21	10.97
	*qTSFSA07*	E2	A07	87	1200-1201	79668425.5-80303499.5	3.58	6.24	-14.27
	*qTSFSB06*	E2	B06	94	3330-3331	147741044-148463751	5.11	8.93	-17.60
	*qTSFSB09*	E3	B09	83	4199-4200	157681437.5-158674261.5	2.53	5.55	-10.23
SSC(DMS)	*qSSDMSA06*	E3	A06	93	1064-1065	113685402-114389748.5	4.40	8.65	9.26
	*qSSDMSA06.1*	E1	A06	96	1066-1067	115079324.5-115729525	17.35	19.87	15.16
		E2	A06	95	1066-1067	115079324.5-115729525	17.05	18.91	12.55
		E4	A06	95	1066-1067	115079324.5-115729525	5.85	9.29	8.87
	*qSSDMSB02*	E3	B02	1	2020-2021	8339151.5-8869640.5	4.32	8.58	-9.02
	*qSSDMSB05*	E4	B05	23	2750-2751	29308615-29722104	4.47	6.40	7.06
	*qSSDMSB06*	E1	B06	97	3330-3331	147741044-148463751	14.67	16.46	-13.23
		E2	B06	97	3330-3331	147741044-148463751	15.48	16.97	-11.46
		E3	B06	96	3330-3331	147741044-148463751	5.46	11.62	-10.31
		E4	B06	96	3330-3331	147741044-148463751	10.09	16.67	-11.99
	*qSSDMSB07*	E4	B07	26	3339-3340	5739088.5-6421542	3.63	5.17	-6.43
	*qSSDMSB07.1*	E1	B07	73	3606-3607	132442678.5-133025444.5	3.49	3.66	-6.13
	*qSSDMSB08*	E4	B08	78	3906-3907	133083267-133817207.5	2.83	4.46	5.89
SSC(FS)	*qSSFSA06*	E3	A06	97	1066-1067	115079324.5-115729525	4.07	6.83	7.70
	*qSSFSB03*	E2	B03	38	2245-2246	12249748.5-12901841	2.76	4.99	5.21
	*qSSFSB06*	E2	B06	93	3329-3330	146842409.5-147741044	5.56	10.15	-7.67
	*qSSFSB06.1*	E3	B06	94	3330-3331	147741044-148463751	3.28	7.72	-6.50
	*qSSFSB07*	E3	B07	59	3601-3602	129436272-130015127.5	3.04	6.86	-6.03
SC(DMS)	*qSDMSA04*	E2	A04	44	650-652	82906761-84376513	3.71	6.56	-0.79
	*qSDMSA06*	E1	A06	95	1066-1067	115079324.5-115729525	12.25	15.52	6.71
		E2	A06	94	1066-1067	115079324.5-115729525	4.35	7.22	3.70
		E4	A06	95	1066-1067	115079324.5-115729525	3.41	5.85	7.22
	*qSDMSA07*	E1	A07	65	1179-1181	67339371-68712293.5	3.09	3.31	-2.93
	*qSDMSB03*	E2	B03	32	2240-2241	9310510.5-9780019.5	2.98	5.89	-0.41
	*qSDMSB05*	E4	B05	10	2722-2723	13702375-14266029	3.01	13.98	8.98
	*qSDMSB06*	E4	B06	92	3329-3330	146842409.5-147741044	4.52	18.14	-10.86
	*qSDMSB06.2*	E1	B06	96	3330-3331	147741044-148463751	15.08	19.46	-7.22
		E3	B06	97	3330-3331	147741044-148463751	4.13	10.33	-1.22
		E4	B06	92	3330-3331	147741044-148463751	15.08	19.46	-0.72
	*qSDMSB08*	E4	B08	75	3906-3907	133083267-133817207.5	3.18	5.54	6.66
	*qSDMSB10*	E4	B10	59	4490-4491	138378396-138918764	2.89	4.66	-6.10
SC(FS)	*qSFSA02*	E3	A02	38	336-337	94332957-95043522	3.44	11.67	-3.67
	*qSFSA07*	E2	A07	33	1075-1076	5006457-5815057.5	3.54	7.14	-3.96

^(1)^: Chr., chromosome; Pos., position; C.I., P.I., physical interval; LOD, Logarithm of odds; Add., additive effect; PVE, phenotypic variation explained.

^(2)^: DMS, dry mature seed; FS, fresh seed. TSC, total sugar content; SSC, soluble sugar content; SC, sucrose content.

^(3)^: Env., environment. E1, Baoding in 2018; E2, Baoding in 2020; E3, Baoding in 2021; E4, Fuxin in 2021.

Eight QTLs were identified for SSC-DMS with 3.66%-19.87% PVE. These were distributed on A06, B02, B05, B06, B07 and B08 (**Table 4**). *qSSDMSA06.1*, detected in E1, E2, and E4 explained 9.29%-19.87% of the phenotypic variance. *qSSDMSB06* detected in E1, E2, E3, and E4 explained 11.62%-16.97% of the variance (**Table 4**).

9 QTLs associated with SC-DMS with 3.31%-19.46% PVE. were mapped on 8 chromosomes including A04, A06, A07, B03, B05, B06, B08 and B10 (**Figure 2**). *qSDMSA06*, detected in E1, E2, and E4, explained 5.85%-15.52%. of the variance. *qSDMSB06.2*, detected in E1, E3, and E4 environments, explained 10.33%-19.46% of the variance. *qSDMSB05*, detected in E4, explained 13.98% of the variance. Donor alleles for *qSDMSB05* were from the male parent ‘Jinonghei3’ (**Table 4**).

Two major chromosomal regions were consistently associated with sugar content. *qTSDMSA06*, *qSSDMSA06.1*, and *qSDMSA06* were co-localized to the same interval (115.08Mb-115.73 Mb), between the flanking markers 1066 - 1067. *qTSDMSB06*, *qSSDMSB06*, and *qSDMSB06.2* were co-localized to the 147.74Mb-148.46Mb region, between flanking markers 3330-3331 (**Table 4**). Additive effects (ranging between 3.70 and 24.69) indicated that the high-sugar content allele for *qA06* was derived from the female parent ‘Silihong’. The additive effect of *qB06* was between -31.58 and -0.72, implying the high-sugar content allele was derived from the male parent ‘Jinonghei3’. Moreover, these QTLs were detected in several environments with PVE values ranging from 7.22 to 19.46 (**Table 4**).

#### 3.2.2 QTLs associated with sugar content in FS

A total of 11 QTLs associated with sugar content in FS across two environments were located on chromosomes A02, A05, A06, A07, A09, B03, B06, and B07, explaining 5.55-11.67% of the variance. 4 QTLs associated with TSC-FS were detected in four chromosomes, explaining 5.55-8.93% of the variance (**Table 4**). Among these, the state major QTL (*qTSFSB06*) explained 8.93% of the variation and co-localized with the major QTL region identified for TSC-DMS (**Figure 2**). For SSC-FS, 5 QTLs were detected in four chromosomes, explaining 4.99-10.15% of the variation. Stable major QTLs (*qSSFSA06, qSSFSB06*) explained 6.83-10.15% of the PVE and overlapped with the stable QTL region detected for SSC-DMS. For SC-FS, 2 QTLs were detected in chromosomes A02 and A07, explaining 7.14%–11.67% of the variation. These two SC-FS loci appeared to be FS-specific loci, and donor alleles conferring higher sugar content were contributed by’Jinonghei3’ (**Table 4**).

### 3.3 Candidate gene mining in the major QTL region on chromosome B06

**Figure 3:**
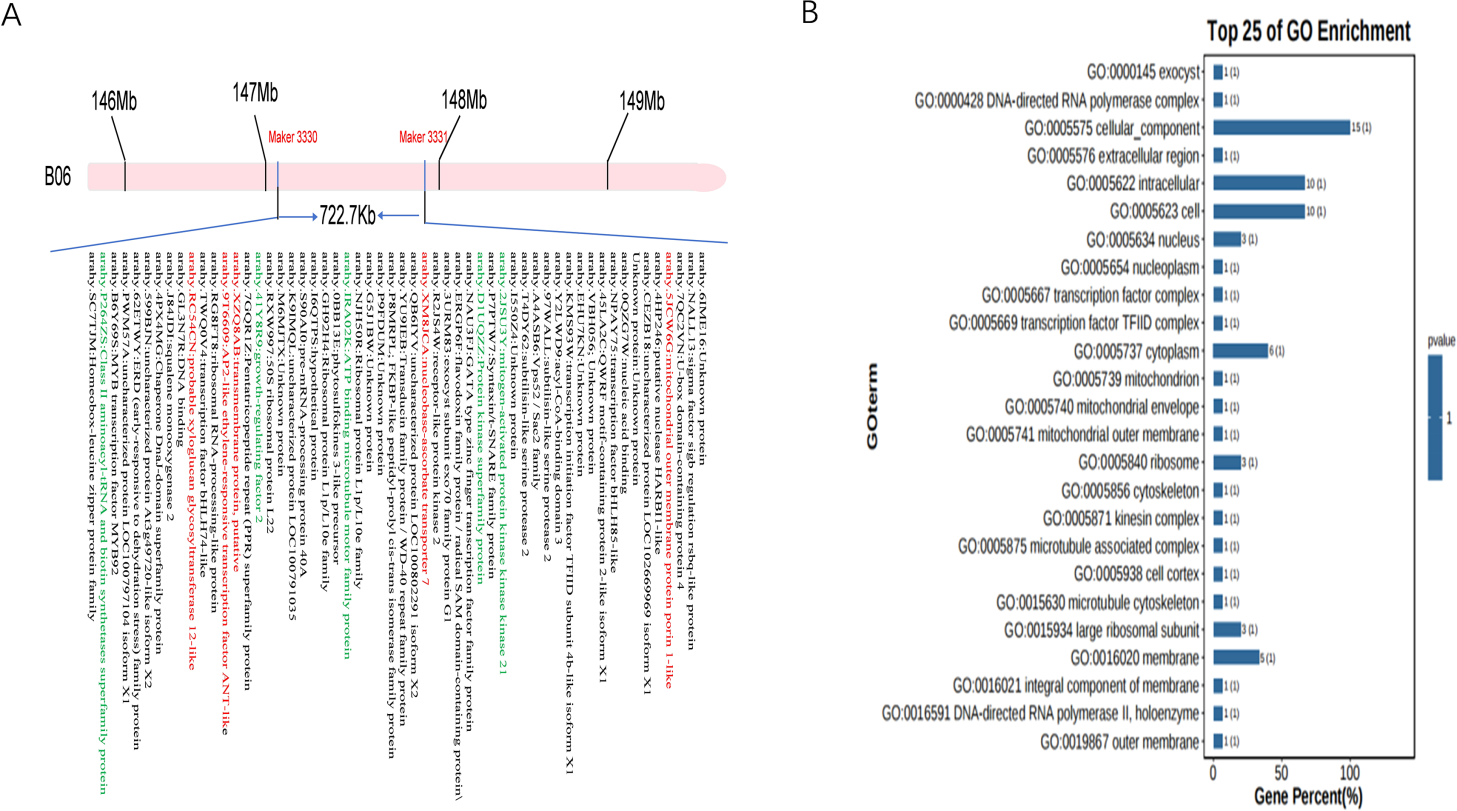
Genes located within the *qB06* region **(A)** and their corresponding metabolic pathways **(B)**.

A co-localized region between markers 3330–3331 on chromosome B06 with PVE values of 7.72% -19.46% was associated with TSC, SSC, and SC in DMS, as well as TSC and SSC in FS. The co-localized region was designated *qSB06*. To further investigate the genetic basis of sugar content in peanut seeds, genes within the *qSB06* interval were examined. The genome of Tifrunner used as the reference genome (Bhat et al., 2023). *qSB06* spanned approximately 722.71kb interval and contained 56 genes (**Figure 3A**). Resequencing of the two parental lines revealed 319 SNPs within this region, including nine within coding sequences. Among these, 1 SNP resulted in a synonymous mutation, 5 SNPs were missense mutations, and 1 SNP was missing (**Table 5**). Gene Ontology (GO) enrichment analysis indicated that most genes were related to cellular components (**Figure 3B**), with 5 genes involved in ATP binding. Among them, *arahy.B6Y69S* encodes the MYB transcription factor MYB92; *Arahy.XM8JCA* encodes an ascorbic acid transporter protein involved in transmembrane transport. *Arahy.3URM83* and *arahy.P7PTW7* encode exo70 family proteins G1 and Syntaxin/t-SNARE family proteins, respectively, involved in vesicle transport.

**Table 5:** Nucleotide types of SNPs located in candidate genes.

Gene name	Gene position	Position	Silihong	Jinonghei3	Function annotation
*arahy.9T6609*	Arahy.16:147880368.147885613	147884905	G	T	AP2-like ethylene-responsive transcription factor ANT-like
*arahy.XZQ8AB*	Arahy.16:147904085.147906280	147904280	A	G	transmembrane protein, putative
*arahy.XM8JCA*	Arahy.16:148133539.148138098	148135236	A	G	nucleobase-ascorbate transporter 7
*arahy.D1UQZZ*	Arahy.16:148195099.148204534	148200131	A	G	ATP binding
*arahy.B6Y69S*	Arahy.16:147733808.147736012	147735746	G	C	MYB transcription factor MYB92
		147735758	G	C	
*arahy.3URM83*	Arahy.16:148166564.148170473	148167024	C	A	exocyst subunit exo70 family protein G1
*arahy.P7PTW7*	Arahy.16:148209848.148213200	148210573	N^(1)^	T	Syntaxin/t-SNARE family protein

^(1)^: N indicates missing.

### 3.4 Expression analysis of candidate genes within the *qSB06* interval during seed drying and maturation

**Figure 4:**
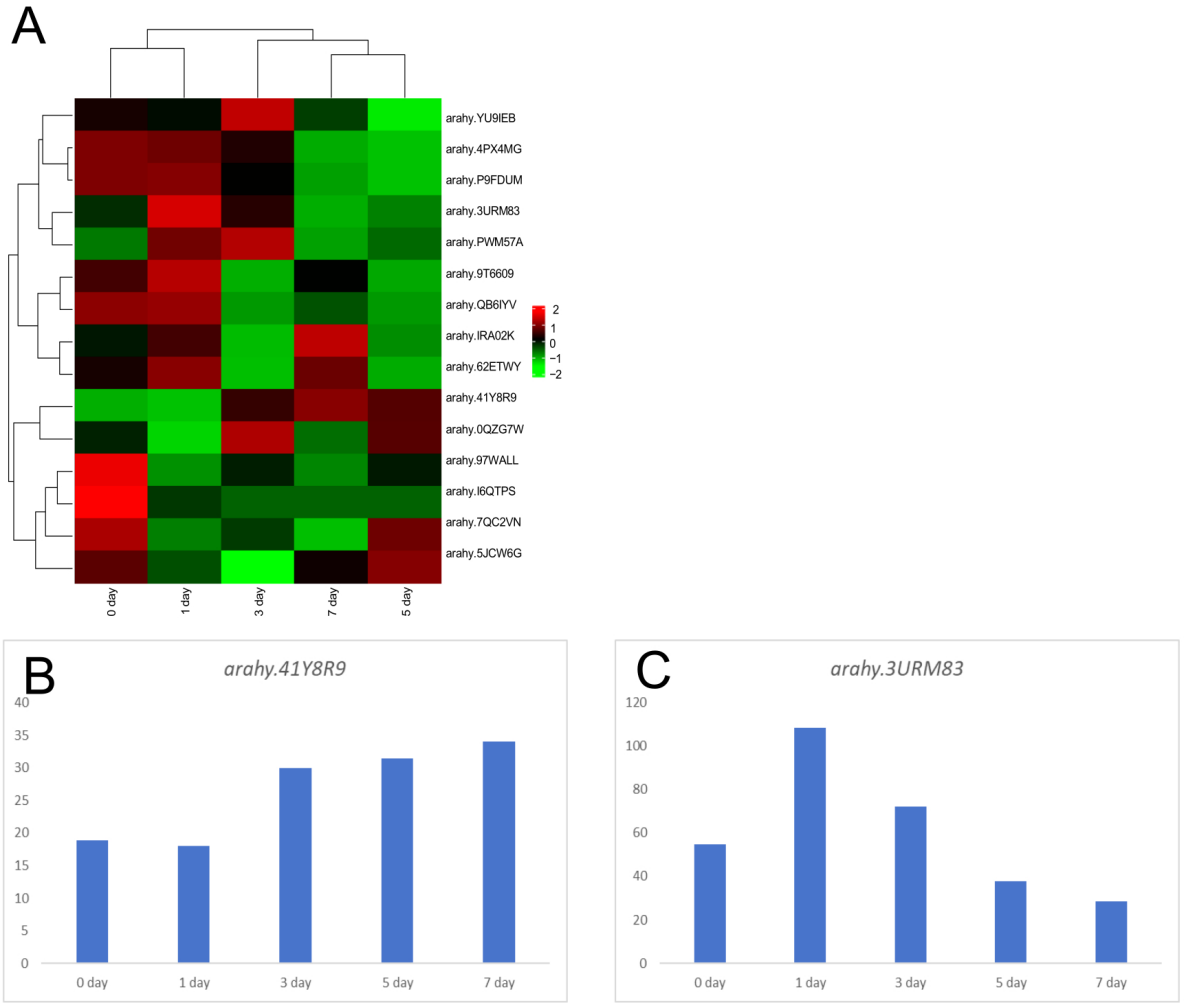
Transcriptome analysis of candidate genes in the *qB06* interval **(A)** Expression profiles of candidate sugar-related genes across five Silihong drying stages; **(B)** Expression levels of *arahy.41Y8R9* in 5 periods; **(C)** Expression levels of *arahy.3URM83* in 5 periods.

The candidate genes within the *qSB06* interval, defined as *arahy.3URM83*, *arahy. 41Y8R9*, and *arahy.P7PTW7*, were tightly associated with sugar synthesis. *Arahy.3URM83*, which encodes an exo70 family proteins of the extracellular vesicle subunit, exhibited an expression pattern that increased initially and then declined during the drying and maturation stages in both parental lines (**Figure 4**). *Arahy.41Y8R9*, encoding growth regulatory factor, showed the opposite expression trend compared to *arahy.3URM83* during maturation, decreasing initially and subsequently increasing (**Figure 4**). Therefore, it can be inferred that this gene is also involved in sugar biosynthesis in peanuts and positively regulates sugar synthesis and accumulation. *arahy. P7PTW7* is a Syntaxin/t-SNARE family protein involved in vesicle-mediated transport; however, in the transcriptome analysis, it showed no significant changes between the two parents (**Figure 4**).

### 3.5 QTL validation using KASP markers

**Figure 5:**
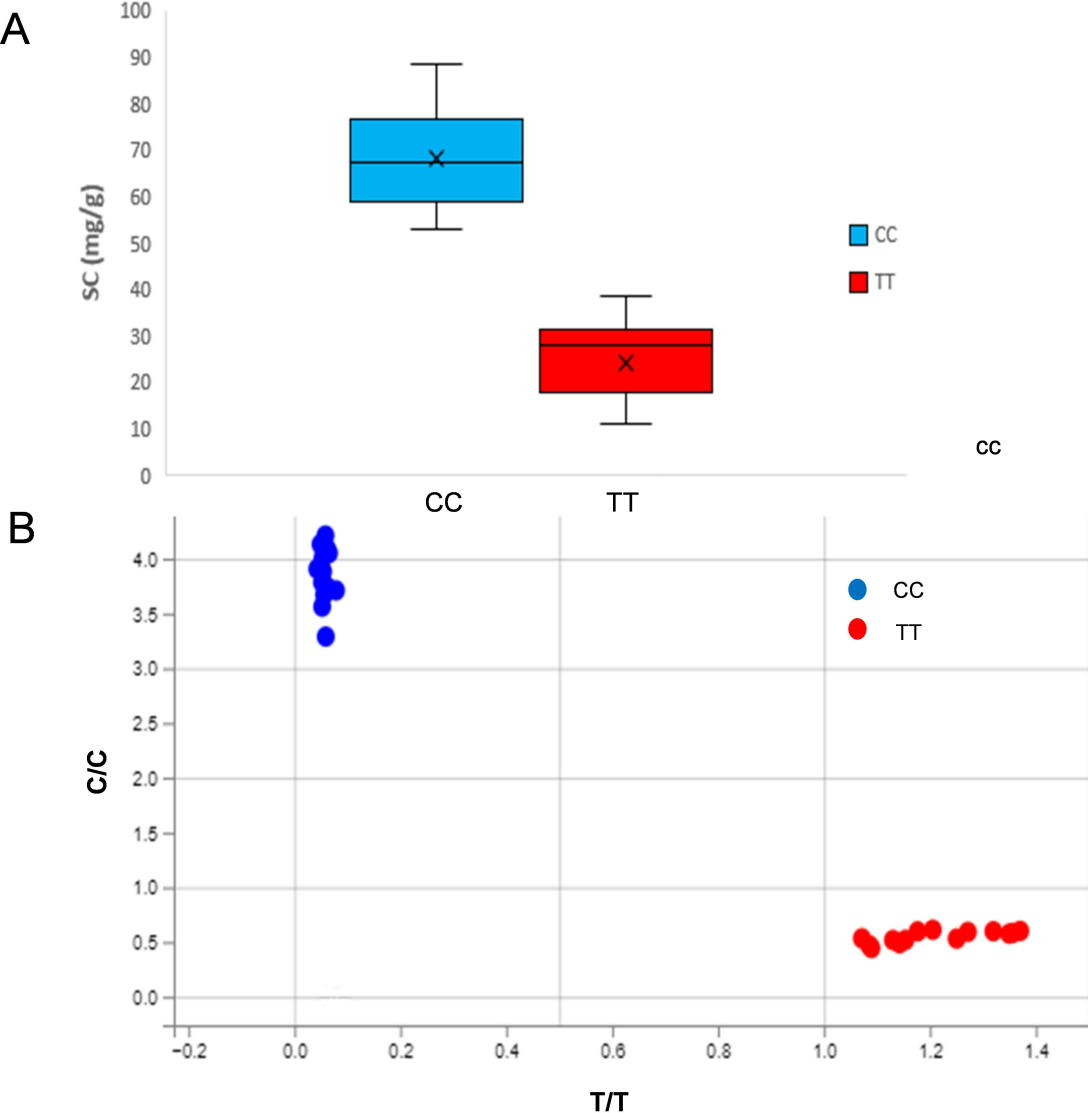
KASP-SC genotyping results and their performance in extreme peanut materials. **(B)** The blue clustered points represent the CC genotype, and the red clustered points represent the TT genotype. **(A)** Box plot of Sucrose content (mg/g) in peanut accessions with the CC and TT genotypes. **(B)** Allele discrimination plot from KASP genotyping of peanut accessions.

KASP markers developed based on the *arahy.PKWB63* within the qB06 interval (physical interval 147.74Mb-148.46Mb, marker interval 3330-3331) were used to validate the association between genotype and sucrose content. Results showed that genotypes carrying the CC allele exhibited significantly higher sucrose content than those with the TT genotype (**Figure 5**). The genotypes of each material and the statistical results of sucrose content are shown in **Table 6**. For the materials with the genotype CC, SC ranged from 53.41mg/g to 88.52mg/g, with an average of 68.35mg/g. For the material with the genotype TT, SC ranged from 11.04mg/g to 38.78mg/g, with an average of 24.63mg/g (**Table 6**). The SC of the materials with the CC genotype was significantly different from that of materials with the TT genotype, and the genotype–phenotype correspondence was consistent. These results indicate that the developed KASPs can effectively screen TSC, SSC, and SC in peanuts.

**Table 6:** Statistical results of genotypes and sucrose content in peanut kernels.

Material number	Genotype	Sucrose content (mg/g)
R-158	C/C	81.11
R-196	C/C	79.21
R-015	C/C	88.52
R-121	C/C	58.23
R-159	C/C	57.26
R-140	C/C	85.63
R-072	C/C	66.84
R-130	C/C	63.42
R-028	C/C	62.14
R-218	C/C	53.41
R-043	C/C	70.75
R-182	C/C	68.31
R-076	C/C	55.47
Funingliyang	C/C	69.45
Jingxianyiwohou	C/C	66.05
Daminglianhua	C/C	67.85
Xinledabacha	T/T	31.55
Pingshanzhonglihuasheng	T/T	12.05
Dalihuasheng	T/T	31.69
Funingxiaozili	T/T	18.25
Damingdayanghuasheng	T/T	20.33
Lulongxiaohuasheng	T/T	28.42
Gaoyiyiwohou	T/T	18.63
Ningjinxiaohuasheng	T/T	17.02
Nongzhanduoli	T/T	31.32
Wenanyiwohou	T/T	38.78
Jiaoheyiwohou	T/T	17.93
Qingyuanyiwohou	T/T	11.04
Funing-Dali	T/T	32.24
Shuluhuasheng	T/T	29.11
Baodingpaman	T/T	28.15
Huayu23	T/T	27.62

## 4 Discussion

### 4.1 The impact of the environment on sugar content

The sugar content in peanut is substantially influenced by environmental factors such as temperature, light, and soil. Specifically, low-temperature environments in cold regions have positively affected sugar accumulation in peanut seeds (Wang et al., 2021). Furthermore, extending light exposure can significantly increase the accumulation of photosynthetic products in leaves, thereby supplying more substrates for sugar synthesis in the seeds (Chen et al., 2020). Soil potassium enrichment also contributes positively, leading to a significant increase in seed sugar content (Abo El-Kasem et al., 2025). Consequently, peanuts cultivated in cold regions typically exhibit higher sugar levels.

Despite experiencing lower temperatures and more favorable light conditions during kernel maturation than Baoding, Fuxin consistently showed lower TSC, SSC, and SC in fresh peanut seeds. This indicates that the relationship between these environmental factors and sugar accumulation in peanuts is complex and may involve other influencing variables. Soil conditions may exert a greater influence than temperature and light. Future studies should integrate meteorological conditions, soil, and other factors to clarify the environmental factors that affect sugar content and identify the differential responses of peanut germplasm with varying sugar levels to temperature and light. This will also provide a theoretical basis for region-specific peanut cultivation aimed at optimizing sugar content.

Interestingly, while lines with low TSC, SSC, and SC of DMS in Fuxin exhibited higher levels than in Baoding, those with higher sugar content displayed the opposite trend. Previous studies have shown that the drying process affects the quality of peanuts and involves genotypic differences (Deng et al., 2025). According to the historical meteorological data, temperatures in Fuxin are lower than those in Baoding during mid-to-late September. During this period of peanut dehydration and drying, the physiological and biochemical activity inside the seeds is vigorous, and it is the key period for sugars to be converted into storage substances such as oil and protein. Low temperature reduces the physiological and biochemical metabolism rate, and the conversion process is hindered. This may be the reason why the DMS sugar content in Fuxin was found to be higher than that in Baoding. Germplasm with high sugar content may be predominantly affected by genetic factors and less by environmental factors. The genetic differences in sugar content during the drying period warrant further investigation.

### 4.2 The effect of the drying process on sugar content

The sugar content of FS is mainly influenced by the expression of metabolic genes active during different stages of seed development. The sugar content of DMS is not only affected by the expression of genes related to each developmental stage, but also by the expression of genes during the drying process. The results of this study demonstrated that sugar content in both FS and DMS is genotype-dependent. DMS represents the physiological maturation of FS after drying; therefore, the QTLs affecting DMS sugar content are more complex. This study conducted QTL analysis on the sugar content of FS and DMS using an RIL population. The *qSB06* affecting SC-DMS, TFC, and SSC in both FS and DMS was a key locus for sugar synthesis. The QTLs of SC-FS differed completely from those in DMS, likely reflecting the distinct physiological role of sucrose in the source–sink dynamics of plants. Sucrose is a product of photosynthesis and the principal transported carbohydrate. After reaching the sink, it can be hydrolyzed into hexoses, which enter the tricarboxylic acid pathway for protein and oil conversion. It may also be converted into polysaccharides such as cellulose and starch (Xu et al., 2025). The final content in FS is therefore largely determined by the genotype. Future research should further dissect the functional differences among QTLs in FS and DMS in order to clarify the key sugar metabolism pathways in FS and DMS.

### 4.3 Novel QTLs identified for sugar content in peanut

Recent studies have explored sugar content traits in mature peanut kernels, using QTL mapping and GWAS analyses to explore the regions and genes related to sugar content traits in peanuts (Zhang et al., 2023). This study identified 22 and 11 QTLs in DMS and FS, respectively, through QTL mapping. The main QTL locus *qSA06*, located on chromosome A06, associated with total sugar, soluble sugar, and sucrose content, overlaps with the QTL located by Huai et al. (2024), which were associated with sucrose and oil content. The other major QTL, *qSB06*, identified in this study, is a newly identified locus associated with total sugar content, soluble sugar content, and sucrose content, and has been detected in DMS and FS across multiple environments. This interval includes a 722 kb region and 56 genes. A major QTL linked to SC was identified in FS, located on chromosome A02, explaining11.67% of the phenotypic variation. Collectively, these QTLs provide valuable targets for breeders to simultaneously or separately optimize sugar content in FS and DMS.

### 4.4 Candidate genes in the major QTL region on chromosome B06

Although the major QTL region on chromosome B06 spanned a relatively large physical interval (~0.72 Mb), candidate genes could still be preliminarily identified. Using reference genome annotations and existing transcriptome data (Wang et al., 2024), three genes (*arahy.3URM83*, *arahy.41Y8R9*, *arahy.P7PTW7*) were identified as potential candidate genes influencing sugar content. *Arahy.3URM83* encodes an exo70 family protein of the outer capsule subunit, and its expression pattern during seed drying exhibits an initial increase followed by a decrease. Dong et al. (2023) identified and performed a genome-wide characterization of the cotton *EXO70* gene family, and found that *EXO70* in cotton is related to fiber quality, and upland cotton with a greater *EXCO70* gene copy number is more sensitive to environmental stress. Wang et al. (2016) found that silencing certain *EXCO70* genes in soybean could inhibit the formation of root nodules and promote premature aging of leaves. Overexpression of *EXCO70* in *Arabidopsis thaliana* revealed that it could affect leaf and flower development. Therefore, it is speculated that it plays an important role in growth and development in leguminous plants by affecting protein and nutrient transport in vesicles. Homologous genes have also been implicated in the negative regulation of sucrose content.

*Arahy.41Y8R9* is a growth regulatory factor (GRF) gene whose expression level during peanut drying decreases initially and then increases, opposite to *arahy.3URM83.* The resequencing results revealed allelic variation between the parents, with a C-to-A substitution at the 460th base in Silihong. Zhao et al. (2019) identified the *GRF* gene family in cultivated peanuts, which were distributed on 20 chromosomes. The expression levels of *AhGRF5a* and *AhGRF5b* genes in pod tissues are higher than those in root, leaf, and stem tissues. Multiple genes of the peanut *GRF* family were found to be involved in grain growth and development. Therefore, it can be inferred that this gene may positively regulate sugar accumulation.

*Arahy.P7PTW7* is annotated as a Syntaxin/t-SNARE family protein involved in vesicle-mediated transport. Rui et al. (2022) conducted functional studies on the *Arabidopsis thaliana* T-SNARE protein *SYP31/SYP32* gene and found that this gene is involved in regulating COPI vesicle-mediated reverse transport and maintaining Golgi apparatus morphology, and plays a role in the development of *Arabidopsis* male gametophytes. Zhang et al. (2016) identified and examined the biological function of the SNARE protein *Fg*Vam7 binding protein in wheat, and found that the SNARE protein *Fg*Pep12 participates in regulating the growth, development, and pathogenicity of *Fusarium graminearum* by participating in vesicle transport. In this study, a base deletion at position 725 was identified in Jinonghei3. The above three genes are closely associated with the transportation and storage of sugars and exhibit sequence variation between the parents. The specific genetic mechanisms involved in regulating sugar synthesis and transport in peanuts need to be further explored.

## 5 Conclusion

In this study, 22 and 11 QTLs related to sugar content in DMS and FS were identified respectively through QTL mapping. Among these, *qA06* (physical location 115.08-115.73 Mb) and *qB06* (physical location 147.74-148.46 Mb) were two major co-localized QTLs identified in multiple environments. KASP markers based on the *arahy.PKWB63* were developed for *qB06* to support marker-assisted selection. Candidate genes (*arahy.3URM83, arahy.41Y8R9, arahy.P7PTW7*) linked to sugar metabolism were identified, pending functional validation. These findings provide a theoretical basis for peanut breeding aimed at improving sugar content, flavor, and industrial quality.

## Data Availability

The datasets presented in this study can be found in online repositories. The names of the repository/repositories and accession number(s) can be found in the article/supplementary material.
